# The Development of Innovated Complex Process for Treatment of Old Flotation Tailings of Copper-Zinc Sulfide Ore

**DOI:** 10.3390/molecules29071550

**Published:** 2024-03-29

**Authors:** Khussain Valiyev, Aliya Bugubaeva, Aleksandra Nechaeva, Alena Artykova, Vitaliy Melamud, Devard Stom, Anna Boduen, Aleksandr Bulaev

**Affiliations:** 1 Research Institute of Applied Biotechnology, Akhmet Baitursynuly Kostanay Regional University, 47A Baitursynov Str., Kostanay 110000, Kazakhstan; v-gulnar-abay@mail.ru; 2 Winogradsky Institute of Microbiology, Research Center of Biotechnology, Russian Academy of Sciences, 7/2 60-letiya Oktyabrya Ave., 117312 Moscow, Russia; nechaevasasha709@gmail.com (A.N.); alena.artykov@gmail.com (A.A.); vmelamud.inmi@yandex.ru (V.M.); 3 Baikal Museum SB RAS, 1 Akademicheskaya Str., 664520 Listvyanka, Russia; stomd@mail.ru; 4 Faculty of Biology and Soil Studies, Irkutsk State University, 1 Karla Marksa Str., 664003 Irkutsk, Russia; 5 RIVS Group of Companies, 11A Zheleznovodskaya Str., 199155 Saint Petersburg, Russia; a_boduen@rivs.ru

**Keywords:** sulfide ores, metallurgical wastes, old flotation tailings, acid leaching, copper, zinc, gold

## Abstract

The possibility of selective Cu and Zn leaching from the sample of old pyrite tailings, which is one of the most widespread types of solid waste forming during non-ferrous metal production, using sulfuric acid solutions and water was studied. It was shown that water leaching provided selective extraction of Cu and Zn and comparatively low iron ion extraction. At the same time, acid leaching provided the obtainment of pregnant solutions with high ferric ion concentration, which can be used for oxidative leaching of substandard copper concentrates. Water and acid leaching also provided increased Au recovery by cyanidation. The results suggest that acid leaching can be an effective approach for processing old flotation tailings, which allows the extraction of base metals from these wastes and treating flotation tailings for subsequent cyanidation. Effective flotation treatment methods should also provide environmental load reduction, which is caused by the long-term storage of metal-bearing wastes.

## 1. Introduction

Sulfide ore processing is characterized by significant losses of non-ferrous and precious metals into various wastes, which in turn pose an environmental problem, but also can be considered a technogenic source of metals due to the decline in metal content in ores as well as deficiency of conventional ores [1,2,3,4,5,6,7,8,9,10].

Sulfide ore flotation tailings, especially those containing large amounts of pyrite as well as other sulfide minerals (containing different non-ferrous metals), are sources of acid mine drainage (AMD) due to the complex process of sulfide oxidation via biotic or abiotic processes induced by water and oxygen availability. Sulfide oxidation results in the formation of secondary minerals, such as sulfates (poitevinite, melanterite, gypsum), hydroxides (goethite), hydroxysulfates (jarosite, alunite), carbonates (malachite, azurite, smithsonite, siderite), and arsenates (scorodite) and release of metal cations and sulfates into the liquid phase [7,9,10,11,12,13,14,15,16].

Thus, in most cases, old sulfide flotation tailings are polymineralic aggregates containing various base metals. These metals can be released into the liquid phase during the storage, which is harmful or potentially harmful to the environment, due to the migration of various released ions possessing long-term harmful effects in areas of tailing disposal. These harmful effects were noted in different regions with various climate conditions [9,13,14,15,16,17,18,19,20].

The base and precious metal contents in flotation tailings are often comparable to those in the processed sulfide ores. The reserves of metals contained in flotation tailing in some sites may be estimated as significant due to the large volumes of wastes accumulated from decades of exploitation of some mineral deposits [15,17,21]. Therefore, investigations have been carried out in recent decades to estimate the possibility of metal extraction from old sulfide-containing tailings using different approaches, which include hydrometallurgical techniques (acid leaching (with sulfuric, organic, hydrochloric leaching), leaching with sodium chloride, cupric chloride, ammonium salts, different oxidants, cyanidation, column and stirred tank reactor bioleaching [2,8,18,21,22,23,24,25,26,27,28,29], roasting [30] and flotation [3], as well as treatment of AMD formed in tailing disposal areas as source of metals [16]. The results of these studies suggest that valuable metals can be successfully extracted from these wastes and high levels of extraction can be reached, while the specific chemical and mineral composition and high iron content (in the form of pyrite and oxide minerals) result in difficulties in base metal extraction. For example, the high content of iron in tailings leads to high concentrations of ferric and ferrous iron in pregnant solutions, which are produced during acid and bioleaching. It was shown that iron concentration can be an order of a magnitude higher than concentrations of base metals [8,17,31,32] which impedes further extraction of base metals from obtained pregnant solutions. Also, the application of comparatively high-cost reagents and equipment does not allow for the use of some methods on an industrial scale despite the high extraction of metals from flotation tailings [17], as low content of useful components in flotation tailings does not repay high CAPEX and OPEX.

Thus, one of the main problems that should be solved for the successful use of flotation tailings as a source of valuable components is the development of simple and cheap technologies, which provide high metal extraction with low costs and without using expensive reagents and complex equipment. Therefore, the treatment of old flotation tailings, which may be considered an important source of base and noble metals, is a technological issue due to the comparatively low content of valuable metals (for example, copper and zinc) and high content of iron as well as oxide minerals, requiring the development of high-efficient approaches for metal extraction.

The goal of the present work was to develop hydrometallurgical approaches based on acid leaching for selective extraction of base metals and gold from the sample of old flotation tailings of copper-zinc sulfide ore containing copper, zinc, and gold, as well as to evaluate the application of flotation tailings as a source of oxidant for treatment of other minerals’ raw materials.

## 2. Results

### 2.1. Column Acid Leaching and Bioleaching

The concentrations of metals in pregnant solutions of the acid leaching are presented in Table 1. Distilled water provided comparatively high extraction of base metals and the best selectivity.

Recovery of copper and zinc reached about 12–18% and 40–43%. Concentrations of copper, zinc, and total iron in the leachate were 0.2–0.3, 1.30–1.41, and 17.4–37 g/L. The increase in H_2_SO_4_ concentration up to 10% did not lead to a significant increase in base metal extraction (Table 1 and Table 2) but led to a significant increase in iron concentration in the pregnant solutions. 

As distilled water provided optimal results of column leaching, solid residues of water leaching were used for further experiments. At first, the residues were leached with water and then with 10% H_2_SO_4_ in the same way as flotation tailings were leached in the first experiment. The acid leaching of the residue did not result in a significant increase in base metal recovery (Table 3). Leaching of the residues with 10% H_2_SO_4_ provided extraction of 7% of copper and 3% of zinc, respectively. Thus, two-stage acid leaching of the residue did not provide a significant increase in copper and zinc extraction but made it possible to obtain the solution with high ferric iron ion concentration.

**Figure 1:**
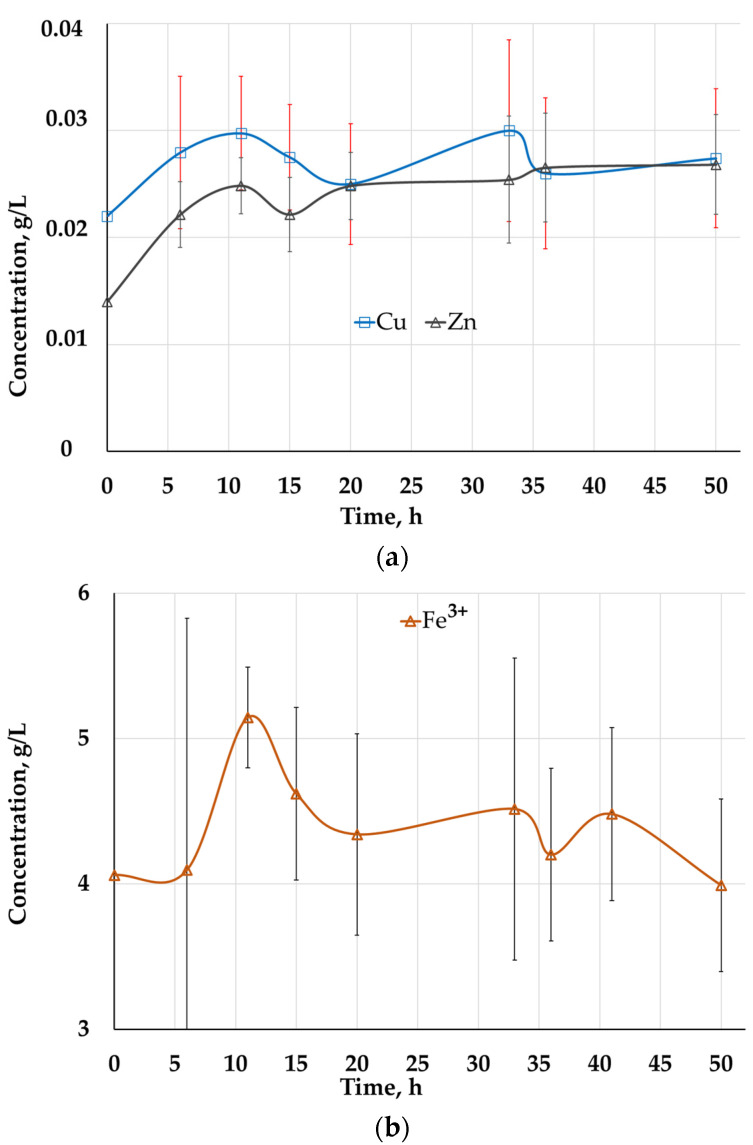
Changes of Zn and Cu (**a**) and Fe^3+^ (**b**) concentrations (g/L) in PLS of bioleaching during 50-day bioleaching.

The solid residues of two-stage leaching with distilled water and 10.0% H_2_SO_4_ were subjected to bioleaching for 50 days which made it possible to additionally extract 2.28 and 1.03% of copper and zinc, respectively. Changes of Zn and Cu and Fe^3+^ concentrations in pregnant leaching solution of bioleaching during 50-day bioleaching are shown in Figure 1. The concentrations of the copper and zinc in the pregnant solutions after 55 days of bioleaching were about 0.0274 and 0.0268 g/L, respectively (Figure 1a). At the same time, ferric iron ion concentration was about 4 g/L (ferrous iron concentration was lower detection limit) (Figure 1b). Thus, bioleaching did not yield pregnant solutions suitable for solvent extraction and did not allow significantly increasing base metal extraction.

### 2.2. Agitation Acid Leaching

The concentrations of metals in pregnant solutions and extraction rates of the agitation leaching are presented in Table 4 and Table 5. Distilled water provided high extraction of base metals and the best selectivity. 

**Figure 2:**
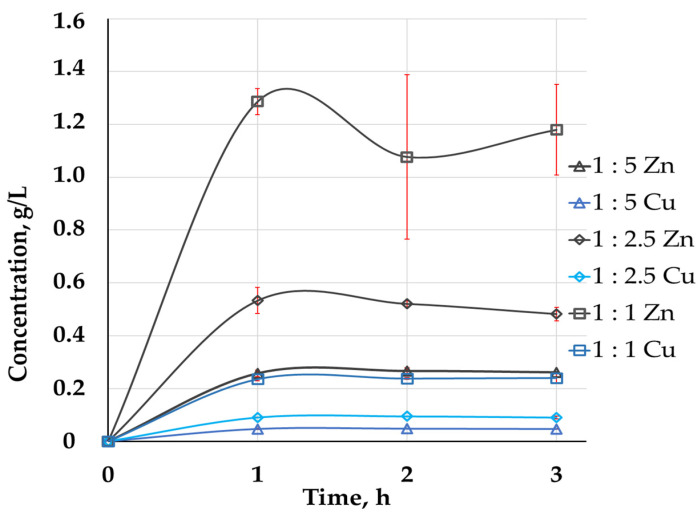
Zn (gray curves) and Cu concentrations (blue curves) (g/L) in PLS of agitation leaching at different pulp densities (S:L 1:5 (triangles), S:L 1:2.5 (rhombi), and S:L 1:1 (squares)).

As distilled water provided optimal results of column leaching, the tendency observed in the experiments with agitation leaching was similar to that observed in the column leaching experiments. At the same time, agitation leaching provided faster metal extraction. Despite the experiments being performed for 3 h, the extraction rates of Cu and Zn were close to maximum values after 1 h of the leaching (Figure 2).

Solid residues of water leaching were used for further experiments to determine the effect of pulp density on the metal extraction (Table 6 and Table 7, Figure 2). It was shown that the increase in the pulp density did not lead to a decrease in copper and zinc extraction. 

To determine the effect of sodium chloride on metal extraction, agitation leaching was performed using 1 M NaCl solution (Table 8). The comparison of the results of the agitation leaching with distilled water and 1 M NaCl solution demonstrated that copper and zinc concentrations did not differ significantly. At the same time, 24-h leaching did not provide additional metal extraction in comparison to 3-h leaching.

Since the increase in pulp density did not lead to a decrease in metal extraction, further experiments were performed at S:L 1:1 to obtain the solution with higher copper and zinc concentrations. At first, the residues were leached with water and then with 5% H_2_SO_4_ in the same way as flotation tailings were leached in the first experiment. Then, 5% H_2_SO_4_ was used to leach water leaching residue twice to increase the content of iron, copper, and zinc in the pregnant solution (II and III stages, Table 9). The acid leaching of the residue did not result in a significant increase in base metal recovery (Table 9). At the same time, the solution with a comparatively high content of ferric iron ions was obtained.

Thus, similar patterns were observed in experiments with column and agitation leaching. In the first stage, water and sulfuric acid solution provided comparatively high copper and zinc extraction, while the increase in sulfuric acid concentration resulted in the increase in Fe^3+^ and Fe^2+^ concentrations and did not allow a significant increase in Cu and Zn extraction. In the second stage, pregnant leaching solutions with high concentrations of Fe^3+^ and Fe^2+^ ions were obtained using 5 and 10% sulfuric acid. The results obtained may be explained by the properties of the minerals of the old flotation tailings [12]. Copper and zinc oxide minerals may be dissolved using water and weak sulfuric acid solutions, while ferric iron dissolution depends on acid concentration to a greater extent, therefore Fe^3+^ and Fe^2+^ concentrations depend on sulfuric concentration.

### 2.3. Copper Concentrate Leaching

Copper extraction in the experiment with alkaline sulfide leaching (ASL) residue was significantly higher than that in the experiment with the concentrate. At the same time, Zn extraction was lower during ASL residue leaching in comparison to the concentrate leaching (Table 10 and Table 11).

The results obtained demonstrated that ASL pretreatment of the concentrate containing tennantite led to a significant increase in copper extraction. This may be explained by the transformation of tennantite which is comparatively refractory to oxidative leaching [33] into copper sulfides, which are readily leachable. In our previous work, we demonstrated that preliminary ASL leaching provided higher copper extraction from the concentrate studied by bioleaching. In the present work, we performed oxidative leaching of the concentrate and ASL residue using Fe^3+^ solution. Since bioleaching and ferric leaching are based on similar principles (interaction of minerals with Fe^3+^ ions), a similar result was obtained: ASL treatment of tennantite-containing concentrate led to tennantite transformation and formation of readily leachable copper sulfide, which may be leached in the presence of Fe^3+^ ions.

### 2.4. Cyanidation 

The data on gold recovery from the residues by carbon-in-pulp cyanidation are presented in Table 12. Column leaching with water and two-stage column leaching with water and 10% H_2_SO_4_ as well as two-stage column leaching and bioleaching provided the highest level of gold recovery. Agitation leaching also increased the recovery of gold. It is likely that leaching promoted the removal of oxide minerals which resulted in an increase in gold extraction level as these minerals can coat a surface of gold particles and limit the access of cyanide solution [34] and their dissolution may liberate part of the gold. Bioleaching did not provide a significant increase in the gold extraction rate. This may be explained by the high pyrite content in the sample of tailings (Section 4.1). Acid leaching may provide the removal of oxide minerals that in turn lead to partial gold deliberation. At the same time, further bioleaching likely did not provide a high pyrite oxidation extent, which in turn did not allow deliberate gold fraction associated with pyrite.

## 3. Discussion

**Figure 3:**
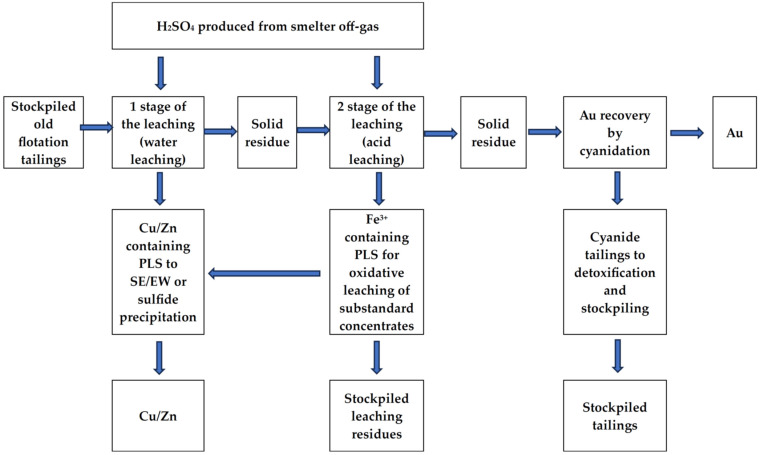
Proposed scheme for old flotation tailing treatment.

The results suggest that acid leaching can be an effective approach for processing old flotation tailings, which allows the extraction of base metals from these wastes and treating flotation tailings for subsequent cyanidation. The here-studied two-stage leaching provided selectivity of base metal extraction and the obtainment of pregnant solutions with low iron concentrations as well as increased gold extraction levels. On the other hand, the second stage of acid leaching provided pregnant solutions with high Fe^3+^ cation content, which may be used as an oxidant for leaching of other mineral raw materials (for example, low-grade concentrates). In the present study, laboratory-scale experiments provided a demonstration of the key process performance parameters; therefore, they should be repeated with other samples of old flotation samples to confirm identified patterns. The results obtained suggest that acid leaching can be a promising strategy for the pretreatment of this type of waste as the studied sample had chemical and mineral composition typical of old flotation tailings of polymetallic ore from deposits located in the Ural region of Russia and other territories. The method proposed does not require high consumption of expensive reagents as well as complex equipment for metal extraction. The method proposed for tailing treatment may be used in existing metallurgical plants located near tailing disposal sites for additional metal production, as well as for oxidant (ferric sulfate solutions) obtaining, which may be used for the treatment of low-grade products (substandard concentrates) since it allows the use of ferric iron formed during long-term disposal of flotation tailings due to oxidative processes [12]. The proposed scheme for old flotation tailing treatment is shown in Figure 3. 

A similar approach was proposed in our previous work, in which the application of an AMD sample as a leaching agent for the treatment of uranium ore samples was used [35]. It should also be noted that the treatment proposed can decrease the environmental load due to the decrease in the easy-leachable fraction of base metals and iron in stockpiled old flotation tailings, which leads to the formation of AMD with high metal content [12].

The results of the present work have some advantages in comparison with previously published results. For example, a comprehensive review [8] summarizes the results of numerous works on acid leaching of flotation tailings. Comparative analysis of the work cited suggests that acid leaching in some cases provided high copper and zinc extraction from the samples of flotation tailings, but most of the works performed were faced with the problem of low selectivity of iron, copper and zinc extraction. At the same time, some of the leaching procedures performed required the application of high concentrations of leaching agents, as well as high temperatures, which suggests the low economic efficiency of some processes proposed [8]. In the works [22,23,26], high rates of Pb, Cu, and Zn extraction from the samples of flotation tailings, as well as high selectivity of metal extraction, were reached using leaching with NaCl and CuCl_2_. Despite the methods proposed providing high metal extraction, they require high consumption of reagents, as well as providing pregnant solutions with high chloride content, which may impede liquid metal extraction and result in equipment corrosion. High copper extraction was shown in the work [28] using the leaching with different ammonium salts, which in turn requires high consumption of reagents. In the work [29], different inorganic and organic acids were used for Fe, Pb, Cu, and Zn extraction from the samples of flotation tailings. In this case, leaching at high temperatures and the use of a wide range of reagents were proposed. In the work [36], STR bioleaching was used for the treatment of flotation tailings samples. As STR bioleaching is usually used for processing of high-grade refractory gold-bearing concentrates [37], which provide the profitability of biooxidation at an industrial scale, treatment of flotation tailings with low content of valuable components cannot provide enough economic efficiency to use STR bioleaching.

The results, similar to the ones obtained in the present study, were obtained in the work [23]. In this case, sulfuric acid solutions were used to extract Co and Cu from flotation tailings.

Thus, the comparison of the results obtained in the present work against those obtained in previous publications showed that, in our work, comparatively high Cu, Zn, and Au extraction rates were reached without using expensive reagents, and without using additional oxidants or high temperatures. The main reagent used for metal extraction was sulfuric acid, which is often produced as a by-product of pyrometallurgy due to the necessity of sulfur dioxide removal from waste gasses [38]. Therefore, the method proposed may be used for additional metal production using mining wastes in the sites, where tailing disposal and metallurgical plants are located.

Summarizing the results of the present work, as well as comparing ones with those obtained in previous works, it can be concluded that:

- flotation tailing treatment is an urgent issue due to specific mineral and chemical compositions;
- in recent years, numerous works on the methods for the treatment of old flotation tailings have been published, which leads to the conclusion on the relevance of the study;
- results obtained in the present work provide efficient extraction of non-ferrous metals and gold from solid wastes studied;
- treatment of flotation tailings, one of the most widespread types of solid waste forming during non-ferrous metal production, which has a harmful impact on the environment, not only allows the extraction of additional metals from the wastes but also provides environmental load reduction due to the decrease in leachable fraction of metals in the wastes;
- in comparison to other works, the approach proposed in the present work does not require the application of expensive reagents and equipment, as well as high energy consumption.

The method proposed may be used for the development of innovative industrial technology providing treatment of ore dressing wastes as well as additional metal production. One of the main advantages of the method proposed is the possibility of its introduction in the flow charts of existing metallurgical plants (Figure 3).

To validate the results obtained in the present study, the following steps are required:

- the method proposed should be studied with other tailing samples, since the results of their treatment using the same methods may provide different rates of metal extraction due to the peculiarities of certain tailings samples;
- pilot scale trials are required to validate the results obtained on a laboratory scale as only long-term experiments may provide real economic analysis of the results;
- to evaluate process efficiency, economic feasibility, and environmental consequence of its application, the semi-industrial test should be performed as only scaling may allow the evaluation of the effect of different factors, which may impede the commercialization of the method proposed (water quality, low temperatures, necessity to utilize the wastes produced during tailings leaching).

The main potential advantages and disadvantages of the method proposed are shown in Table 13.

## 4. Materials and Methods

### 4.1. Old Flotation Tailings Sample

**Figure 4:**
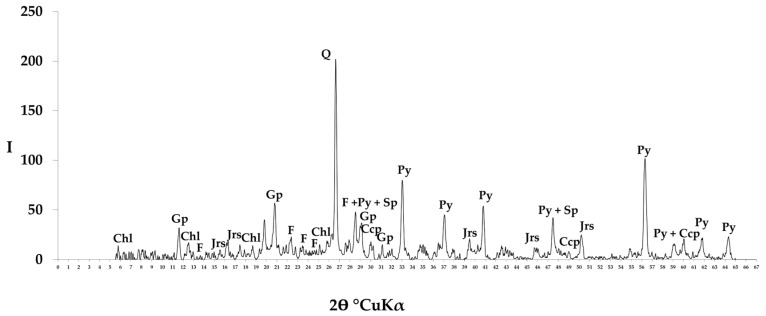
X-ray diffraction pattern of the old pyrite flotation tailings sample obtained using a DRON-2 diffractometer (CuKα, step size 0.02) (Burevestnik, St. Petersburg, Russia). Ccp: chalcopyrite; Chl: chlorite; F: feldspar; Gp: gypsum; Jrs: jarosite; Q: quartz; Py: pyrite; Sp: sphalerite.

A sample of old pyrite flotation tailings obtained at an industrial concentrator was used. The main elements contents and mineral composition of the old pyrite flotation tailings are presented in Table 14 and Table 15 and Figure 4. The sample was milled using an LDI-65 disc eraser (Techoborudovanie, Saint Petersburg, Russia) and then mixed to produce a representative sample of raw material for chemical and mineralogical analyses as well as for the experiments. The particle size distribution had a P95 of 74 μm (i.e., the proportion of particles less than 74 μm in size was 95%).

### 4.2. Column Acid Leaching and Bioleaching

The samples of old pyrite tailings (100 g) were leached in glass columns with perforated bottoms (5 cm in diameter and 20 cm in height) at ambient temperature (~25 °C) with sulfuric acid (Rushim, Moscow, Russia) solutions (100 mL of sulfuric acid solution were used for the treatment of 100 g of each sample of old pyrite tailings). Sulfuric acid solutions with concentrations from 0.5 to 10% (0.5, 1.0, 2.5, 5.0, and 10.0%), as well as distilled water were used for leaching. The percolation rate was not controlled, solid samples were flooded with acid solution and leached once-through. As percolation leaching was not controlled, the duration of the column leaching experiment was also not controlled and the time of leaching was determined by the rate of solution leakage. The content of iron ions, as well as copper and zinc in pregnant leach solutions (PLS) were analyzed. The solid residues of the acid leaching were used in bioleaching and cyanidation experiments.

An indigenous mixed culture of acidophilic chemolithotrophic microorganisms obtained from an AMD sample of pyrite tailings was used for the bioleaching experiments. *Acidithiobacillus* sp., *Leptospirillum ferriphilum*, *Sulfobacillus* sp., *Ferroplasma acidiphilum*, and *Acidiplasma* sp. were identified in microbial population [40]. Representative samples of solid acid leaching residues (100 g) were processed in air-lift percolators (4 cm in diameter and 40 cm in height). Bioleaching was conducted at ambient temperature (20–25 °C) for 50 days to evaluate the possibility of additional metal extraction after acid leaching.

### 4.3. Agitation Acid Leaching

Agitation leaching was carried out using a K.121 bottle agitator (Metallotehnika, Krasnoyarsk, Russia). The effect of leaching process parameters on metal leaching was studied at ambient temperature (~25 °C).

Sulfuric acid solutions (from 0.5 to 10%) or distilled water were used to determine the effect of H_2_SO_4_ concentration on the extraction of metals from old flotation tailings. To determine the effect of sulfuric acid concentration on the metal extraction, the pulp density (solid-to-liquid ratio, S:L) was 1:5 and the leaching time was 3 h.

To determine the effect of pulp density on metal extraction, the pulp densities studied (solid to liquid ratio, S:L) were 1:5, 1:2.5, and 1:1 and the leaching time was 3 h. Distilled water was used in the experiments with different pulp densities.

Since chloride-ion presence may increase the rate of copper and zinc leaching from sulfide minerals, the effect of sodium chloride on the leaching was studied [41]. To determine the effect of sodium chloride on metal extraction, agitation leaching was performed using 1 M NaCl (Rushim, Moscow, Russia) solution. To determine the effect of 1 M NaCl on the metal extraction, the pulp density (solid to liquid ratio, S:L) was 1:5 and the leaching time was 3 and 24 h. Distilled water was used as a control in the experiment with NaCl and results of agitation leaching with distilled water and 1 M NaCl were compared.

### 4.4. Cyanidation

The data on gold recovery from the residues of acid- and bioleaching by carbon-in-pulp cyanidation were used as the criterion to determine the efficiency of the proposed techniques for increase in gold recovery. The extent of gold recovery was determined by carbon-in-pulp cyanidation, which was conducted under the following conditions: pulp density of 40% (*w*/*v*), pH 10.5–11.0 (adjusted using 20% CaO (Rushim, Moscow, Russia), cyanide (NaCN) (Korund-Cyan, Dzerjinsk, Russia) concentration of 2.0 g/L, sorbent content (HyCarb carbon) (HAYCARB PLC, Colombo, Sri Lanka) of 8% (*w*/*v*), and 25 °C using K.121 bottle agitator (Metallotehnika, Krasnoyarsk, Russia). The experiments were performed for 24 h. To determine the degree of gold recovery, leaching and cyanidation residues, as well as sorbent were analyzed for Au content and the recovery rate was calculated (analysis of Au content in solid products was carried out in the analytical laboratory of JSC Regional Analytical Center Mekhanobr Engineering Analyte (Saint-Petersburg, Russia)).

Au extraction was calculated using the formula:

$$\mathrm{Au}\;\mathrm{extraction}\;\left(\%\right)=\frac{\left(\mathrm{Au}\;\mathrm{content}\;\mathrm{in}\;\mathrm{cyanidation}\;\mathrm{residue}\;\times \mathrm{Cyanidation}\;\mathrm{residue}\;\mathrm{yield}\;\left(\%\right)\times 0.01\right)}{\mathrm{Initial}\;\mathrm{Au}\;\mathrm{content}\;\mathrm{in}\;\mathrm{the}\;\mathrm{product}}\;\times 100\%\;$$

### 4.5. Copper Concentrate Leaching

A pregnant solution of the agitation leaching containing ferric iron ions was used to perform oxidative leaching of Cu-Zn concentrate containing 18.1% Cu, 6.2% Zn, and 1.7% As, as well as the product of its alkaline sulfide leaching (ASL) containing 17.8% Cu, 6.4% Zn, and 0.2% As. The main minerals of the concentrate were pyrite (FeS_2_), chalcopyrite (CuFeS_2_), tennantite (Cu_12_As_4_S_13_), and sphalerite (ZnS). ASL concentrate residue was obtained in our previous work by means of the leaching of the concentrate with 3.5 M NaOH and 1.5 M Na_2_S at 95 °C in a stirring tank reactor [33]. ASL allows the removal of arsenic by means of the destruction of tennantite which results in the formation of dissolved thioarsenite and CuS and CuS_2_ in solid residues. It was shown that ASL pretreatment of the concentrate containing tennantite led to a significant increase in copper extraction by bioleaching as tennantite is a high-refractory mineral in terms of its oxidative leaching [33]. In the present work, we performed oxidative leaching of the concentrate and ASL residue using a pregnant solution of old flotation tailings leaching to evaluate the possibility of using old flotation tailings as the source of valuable oxidant. The leaching was performed at 90 °C for 5 h, and pulp density (S:L) was 1:100. Leaching performed in 250 mL laboratory reactors with a working volume of 100 mL. TW-2.03 circulating water baths (Elmi, Riga, Latvia) were used to maintain the temperature; RW20 overhead stirrers (IKA, Staufen, Germany) were used for stirring (200 rpm).

### 4.6. Analytical Methods

The values of pH and Eh were measured with a pH-150 MA pH meter–millivoltmeter (Izmeritelnaya Tehnika, Moscow, Russia). The concentrations of Fe^3+^ and Fe^2+^ ions in PLS were determined through reaction with potassium thiocyanate on a KFK-3 photometer (ZOMZ, Russia) at λ = 475 nm. The concentrations of copper and zinc ions in PLS were determined on a Perkin Elmer 3100 flame atomic absorption spectrometer (Perkin Elmer, Waltham, MA, USA). The gold content in the solid phase was measured using a fire assay [42].

### 4.7. Data Processing

Leaching experiments were performed in duplicate. Processing of the results was carried out using the MS 15.0.459.1506 Excel 2013 software (Microsoft, Redmond, WA, USA). Average values (±SD) of the parameters are presented.

## 5. Conclusions

The results suggest that acid leaching can be an effective approach for processing old flotation tailings, which allows the extraction of base metals from these wastes and the treatment of flotation tailings for subsequent cyanidation. Two-stage leaching may provide selectivity of base metal extraction and the obtainment of pregnant solutions with low iron concentrations as well as increase gold extraction level. Our laboratory-scale experiments provided a demonstration of the key process performance parameters and should be repeated with other samples of old flotation samples to confirm identified patterns. Despite a lot of different old flotation samples possessing similar chemical and mineral composition, the results of their treatment using the same methods may provide different rates of metal extractions due to the peculiarities of certain tailings samples. Scaling of the process studied is also required since only pilot scale trials may provide economic analysis and environmental impact assessment of the methods proposed. At the same time, the results obtained suggest that acid leaching can be a promising strategy for the pretreatment of this type of waste as the studied sample had chemical and mineral composition typical of old flotation tailings of polymetallic deposits located in the Ural region of Russia and other territories.

## Figures and Tables

**Table 1:** Concentrations of the metals in pregnant solutions of the column acid leaching with sulfuric acid solutions.

H_2_SO_4_ Concentration, % (*w*/*v*)	Fe^3+^, g/L	∑Fe, g/L	Cu, g/L	Zn, g/L
0 (dist. water)	2.3 ± 0.4	17.4 ± 0.2	0.199 ± 0.06	1.31 ± 0.03
0.5	2.1 ± 0.1	17.8 ± 0.9	0.205 ± 0.01	1.31 ± 0.05
1.0	2.4 ± 0.0	19.2 ± 0.5	0.223 ± 0.001	1.35 ± 0.01
2.5	5.4 ± 0.2	23.8 ± 2.5	0.235 ± 0.049	1.30 ± 0.23
5.0	10.4 ± 2.0	31.5 ± 3.5	0.270 ± 0.000	1.34 ± 0.01
10.0	16.5 ± 0.0	37.0 ± 2.5	0.314 ± 0.021	1.41 ± 0.09

**Table 2:** Copper and zinc extraction in pregnant solutions of the column acid leaching with sulfuric acid solutions.

H_2_SO_4_ Concentration, % (*wt*/*v*)	Extraction, %	
	Cu	Zn
0 (dist. water)	11.6 ± 0.1	40.0 ± 0.9
0.5	11.9 ± 0.1	39.9 ± 1.7
1.0	13.0 ± 0.1	40.9 ± 0.2
2.5	13.7 ± 0.9	39.6 ± 6.8
5.0	15.8 ± 0.0	40.9 ± 0.1
10.0	18.3 ± 0.4	42.9 ± 2.8

**Table 3:** Concentrations of the metals in pregnant solutions of the two-stage column acid leaching with sulfuric acid solutions.

Stage	Fe^3+^, g/L	∑Fe, g/L	Cu, g/L	Zn, g/L
I stage—Dist. water leaching	2.8	19.4	0.21	1.40
II stage—10.0% H_2_SO_4_ leaching	20.6 ± 0.9	27.5 ± 8.0	0.13 ± 0.01	0.11 ± 0.04

**Table 4:** Concentrations of the metals in pregnant solutions of the agitation acid leaching with sulfuric acid solutions (S:L 1:5, 3 h).

H_2_SO_4_ Concentration, % (*w/v*)	Fe^3+^, g/L	∑Fe, g/L	Cu, g/L	Zn, g/L
0 (dist. water)	1.7 ± 0.6	3.9 ± 0.1	0.023 ± 0.004	0.15 ± 0.05
0.5	3.3 ± 2.5	6.9 ± 1.1	0.025 ± 0.004	0.16 ± 0.01
1.0	2.9 ± 1.0	8.3 ± 1.6	0.027 ± 0.008	0.15 ± 0.08
2.5	6.1 ± 0.3	9.7 ± 2.3	0.029 ± 0.003	0.16 ± 0.01
5.0	5.5 ± 0.9	7.6 ± 1.8	0.021 ± 0.000	0.15 ± 0.01
10.0	5.7 ± 0.4	9.4 ± 2.8	0.034 ± 0.013	0.18 ± 0.07

**Table 5:** Copper and zinc extraction in pregnant solutions of the agitation acid leaching with sulfuric acid solutions (S:L 1:5, 3 h).

H_2_SO_4_ Concentration, % (*w*/*v*)	Extraction, %	
	Cu	Zn
0 (dist. water)	9.6 ± 1.8	28.9 ± 9.4
0.5	10.4 ± 1.8	31.4 ± 2.0
1.0	11.2 ± 3.5	29.5 ± 15.9
2.5	12.0 ± 1.2	32.3 ± 2.3
5.0	12.2 ± 0.0	29.0 ± 1.9
10.0	14.2 ± 5.3	35.1 ± 13.7

**Table 6:** Concentrations of the metals in pregnant solutions of the agitation leaching (distilled water, 3 h) at different pulp densities.

S:L	Fe^3+^, g/L	∑Fe, g/L	Cu, g/L	Zn, g/L
1:5	0.8 ± 0.2	3.8 ± 0.0	0.047 ± 0.001	0.261 ± 0.001
1:2.5	1.1 ± 0.2	2.5 ± 0.2	0.090 ± 0.006	0.482 ± 0.026
1:1	2.2 ± 0.25	6.6 ± 2.2	0.240 ± 0.020	1.180 ± 0.171

**Table 7:** Copper and zinc extraction in pregnant solutions of the agitation leaching (distilled water, 3 h) at different pulp densities.

S:L	Extraction, %	
	Cu	Zn
1:5	19.3 ± 0.3	50.2 ± 0.2
1:2.5	18.8 ± 1.2	46.3 ± 2.5
1:1	20.0 ± 1.6	45.4 ± 6.5

**Table 8:** Copper and zinc concentrations in pregnant solutions (g/L) of the agitation leaching (distilled water (control and 1 M NaCl solution, S:L 1:5, 3 and 24 h).

Metal	Variant	Duration, h	
		3	24
Cu	Control	0.049 ± 0.001	0.049 ± 0.002
	1 M NaCl	0.046 ± 0.002	0.047 ± 0.002
Zn	Control	0.28 ± 0.01	0.29 ± 0.01
	1 M NaCl	0.27 ± 0.01	0.28 ± 0.01

**Table 9:** Concentrations of the metals in pregnant solutions of the agitation acid leaching with sulfuric acid solutions (S:L = 1:1, 3 h).

Stage	Fe^3+^, g/L	∑Fe, g/L	Cu, g/L	Zn, g/L
I stage—Dist. water leaching	4.6	16.5	0.26	1.39
II stage—5.0% H_2_SO_4_ leaching	7.0	9.45	0.066	0.142
III stage—5.0% H_2_SO_4_ leaching	8.4	11.2	0.129	0.228

**Table 10:** Concentrations of the metals in pregnant solutions of the oxidative copper concentrate and ASL residue.

Product	Fe^3+^, g/L	∑Fe, g/L	Cu, g/L	Zn, g/L
Concentrate	4.9 ± 0.3	11.6 ± 0.5	0.65 ± 0.03	0.76 ± 0.03
ASL residue	5.3 ± 0.2	11.8 ± 0.2	1.03 ± 0.03	0.56 ± 0.10

**Table 11:** Copper and zinc extraction in pregnant solutions of the oxidative of copper concentrate and ASL residue.

Product	Extraction, %	
	Cu	Zn
Concentrate	29.3 ± 1.6	91.1 ± 4.1
ASL residue	51.4 ± 1.9	55.8 ± 17.1

**Table 12:** Gold extraction from the old flotation tailings and leaching residues by cyanidation.

Product	Extraction, %
Flotation tailings	35 ± 18
Agitation leaching residue I stage	44 ± 2
Agitation leaching residue II stage	39 ± 8
Column acid leaching I stage	50 ± 18
Column acid leaching II stage	45 ± 29
Column acid leaching II stage + bioleaching	48 ± 4

**Table 13:** Potential advantages and disadvantages of the process studied in the present work.

Advantage	Disadvantage
Economical feasibility	
additional metal production comparatively low CAPEX and OPEX due to the simplicity of the method proposed possibility of application of metallurgical by-products (off-gas) and treatment of low-grade concentrates lack of necessity to use high-cost reagents comparatively high rate of metal extraction	CAPEX and OPEX for process commercialization comparatively low metal extraction performance instability due to the difference in chemical and mineral composition of old flotation tailings
Environmental impact	
decrease in the content of leachable fraction of metals in old flotation tailings, as well as the decrease in metal content in formed AMD lack of necessity to use specific reagents, which have hazardous environmental effects	formation of the wastes after acid leaching and cyanidation the necessity to manage formed wastes
Comparison with previous studies [8,22,23,26,27,28,29,36,37,38]	
simplicity and low CAPEX and OPEX possibility to use old flotation tailings as a source of valuable oxidants (ferric solutions) low reagent and energy consumption	comparatively low metal extraction in comparison to the methods of autoclave leaching, bioleaching, oxidants, etc.

**Table 14:** Mass fraction of main elements in the old pyrite flotation tailings determined by using a phase analysis method [39].

Content					
Fe_tot_ *, %	S_tot_ *, %	S_s_ *, %	Cu, %	Zn, %	Au, g/t
29.5	29.2	25.4	0.12	0.26	0.8

* Designations Fe_tot_, S_tot_, S_s_ signify total iron content, total sulfur content, and sulfide sulfur content, respectively.

**Table 15:** Mineral composition of the old pyrite flotation tailings sample according to an XRD analysis performed using a DRON-2 diffractometer (CuKα, step size 0.02) (Burevestnik, St. Petersburg, Russia).

Mineral	Formula	Content (wt %)
Pyrite	FeS_2_	49
Chalcopyrite	CuFeS_2_	<0.5
Sphalerite	ZnS	<0.5
Gypsum	CaSO_4_∙2H_2_O	8
Jarosite	KFe_3_(SO_4_)_2_(OH)_6_	1
Chlorites	(Mg,Fe)_3_(Si,Al)_4_O_10_(OH)_2_·(Mg,Fe)_3_(OH)_6_	6
Feldspars	KAlSi_3_O_8_–NaAlSi_3_O_8_–CaAl_2_Si_2_O_8_	8
Quartz	SiO_2_	29

## Data Availability

The data presented in this study are available on request from the corresponding author. The data are not publicly available as the work does not contain the data, which can be deposited in public databases.
